# Local Anesthesia in Every-Day Conservative Practice

**Published:** 1921-04

**Authors:** Alexander Naismith


### LOCAL ANESTHESIA IN EVERY-DAY CONSERVATIVE PRACTICE

BY ALEXANDER NAISMITH, L.D.S.

This being the first ordinary meeting since you did me the honor of electing me to the chair, I can not allow the opportunity to pass without expressing my appreciation of your action and my sense of the responsibility that honor carries with it. That you have considered me worthy to preside over our deliberations, as so many able and worthy men have done during these last fifty-two years, while it is very gratifying to me, induces an introspective frame of mind which fails to bring self-assurance. One, however, gains some degree of confidence in reflecting that the usefulness of no community depends upon any one person; that in a Society such as this, where the aim of every member is identical, where the progress of the profession is equally dear to all, one may depend upon the loyal support of everyone.

In addressing you tonight, I do so, not imagining that I have anything to say of outstanding originality. Indeed, the application of local anesthesia in all its branches has been treated so fully and scientifically during the last few years, that it would almost seem an impertinence to bring it before you now. In my association with members of our profession, I find, however, that there are those, who for one reason or another, have not made use of it in their every-day conservative work. Having, for a good many years, made regular use of it with great comfort to myself and benefit to my patients, I thought it might be helpful to those if the story was told again by a humble member of the profession who can lay claim to no particular ability, but only to a desire to be helpful.

Possibly the reasons why those have not adopted it are —an exaggerated idea of the difficulties attending its application, or they may be averse to introducing potent drugs into the deeper tissues. To overcome the first difficulty a clear knowledge of the anatomy of the parts dealt with is necessary. One ought to be able to visualize, as it were, the passage of the needle and the anesthetic. By a reasoned consideration of what course ought to be adopted in a particular case, and care in carrying out the various steps of the procedure, there is no reason why it should not prove generally successful. Some practice will be required before one can be reasonably certain of results, but that is necessary to success in every department of our work. The second objection we can more fully respect when we remember the serious effects which often followed the use of cocaine anesthesia in the early days. Fortunately we now have novocaine, which practically fulfils the demands of an ideal anesthetic. Its comparative low toxicity, great and rapid action, and its non-irritating character, together with its ability to withstand being boiled without deterioration, are its outstanding features. Combined with a small proportion of suprarium and in freshly-prepared solution, serious toxic effects are almost entirely absent.

After prevention, our highest dental ideal is the restoration of defective teeth to usefulness, and whatever will help to that end is worthy of our consideration. In local anesthesia we have a means not only of rendering the work less disagreeable to the patient and less trying to the operator, but which conduces to more thorough preparation of cavities, and insures painless and immediate extirpation of exposed or inflamed pulps. By its means the best service of the dentist is brought within the compass of the most timorous, and restorations are possible with its aid which would otherwise be impossible. Especially in treating children does one realize this. With them it seldom fails to give the desired result; the bones are still soft and more vascular than later, there is no reflex disturbance and after-pain is almost unknown. The result is that the natural dread of the dentist is absent, and periodical inspection and treatment become more regular.

We have four methods by which to obtain insensitivity of the teeth: submucosal, peridental, intra-alveolar and regional. Each of these has its particular sphere of usefulness. Each has its advantages and disadvantages. In the submucous method we have to rely upon the minute foramina passing through the hard cortex into the cancellous interior of the bone to convey the anesthetic to the tooth apex. While these foramina are usually present in definite areas, they vary so greatly in number and size in different subjects, being generally more pronounced in the young and almost entirely absent in age (on the buccal surfaces at any rate), that one can not be certain of obtaining full anesthesia even in these areas. When successful, the drug requires about ten minutes to take effect. By the time the small quantity which finds its way through these canals reaches the apex of the tooth it must have become greatly diluted and its effect correspondingly weakened.

The principal areas where these foramina are to be found are: on the free alveolar margin in both jaws; on the mandible, between the root eminences of the incisors and in front of the canine, extending from the alveolar edge about half an inch towards the apices. There is also a very constant and useful cluster in the incisor fossa. On the remainder of the mandible they are almost entirely absent, with the exception of a comparatively large but not constant one on the lingual surface entering the septum between the central and lateral of either side just below the alveolar edge, and a somewhat larger one in the center line immediately over the genial tubercles. These might be made use of. In the maxilla many small foramina are found on the whole buccal surface as far back as the second bicuspid, while the palate is almost cribriform. On the palate, immediately over the free margin and between the central, lateral and canine, there are usually one or two definite canals, and, over the bicuspids and molars, many just at the turn of the vault. In the upper jaw, anesthesia of the pulp can be obtained by this method with a fair degree of certainty as far back as the bicuspids, but in the lower jaw it can not be expected beyond the laterals. The needle used ought to have about three-quarters of an inch free, with a fairly long bevel. It is inserted about one-eighth of an inch from the gum margin, with the bevel towards the alveolar surface, injecting while slowly advancing till about level with the apex of the tooth. In both this and the intra-alveolar method the injection should generally be made, in the upper jaw, anterior to the tooth to be affected, excepting in the case of the central incisors; in the lower jaw, distally in all cases. Exceptions occur where the teeth are crowded, when the procedure must be varied to suit.

Peridental injection may be used for all teeth. The technique is simple, and it is a very satisfactory method in use, as, by it, the anesthetic is forced directly into the peridental membrane at the gingival margin. Provided there is no periodontitis or pyorrhea present, and, if one is satisfied that the gum sulcus can be rendered thoroughly aseptic, it is a safe procedure. It has this advantage, that a short, straight needle only is used. This should be about a quarter of an inch long and fairly stout, as considerable pressure is sometimes required to insinuate it between the tooth and alveolus. The interchangeable style of needle is not satisfactory, as these are apt to slip through the soft metal end and allow the fluid to escape. There is also a degree of movement within the hub attachment which is not conducive to success. Those of the imperial type are to be preferred on account of their rigidity. For this and intra-alveolar injection I use a small bore syringe, holding about fifteen m, the plunger being little over one-eighth of an inch in diameter. The usual leather packing is replaced with asbestos twine impregnated with vaseline, and in place of the leather washer at the nozzle a little tin is melted into the recess usually found there. A little scraping is necessary to obtain a tight seating for the hub. These, packing and washer, last for months of constant use, and rid the syringe of otherwise objectionable features. The whole can be thoroughly sterilized by boiling without injury to the packings.

This peridental anesthesia is particularly useful about the lower molars when it is desired to avoid making use of regional anesthesia, and where the intra-alveolar method is not always suitable. With the first and second molars the injection is best made between the two roots. The point of the needle should have a fairly long bevel, and, with the lumen facing the tooth, is inserted between the tooth and gum at a point just behind the middle of the mesial root. Pressing firmly, slightly backwards and inwards, the needle is forced between the root and alveolus. Considerable pressure is necessary on the plunger to force the anesthetic into the membrane. The small diameter of the piston gives one the necessary pressure with less effort than with a larger syringe. Sometimes it is necessary to make a second injection posterior to the distal root to affect the nerve supply to it. With the third molar the injection is made behind it, as the bone is more vascular there, and, if the roots are massed, it is not possible to effect an entrance as with the other molars. With the upper molars the procedure is much the same, but, occasionally, it is necessary to make a further injection palatally. This is best effected mesially to the palatine root.

Intra-alveolar anesthesia, which is associated in our minds with the name of Parrott, is in my experience the most generally useful method. It combines simplicity with an almost positive certainty of success. There is a directness and definiteness about it which appeals to the practical mind. Parrott makes a preliminary injection into the interdental gingiva to anesthetize the superficial tissues. Having done this he uses a small bur to pierce through the cortex at a point as near the apex of the tooth as possible, avoiding the mobile tissues. Then, with a high-pressure syringe and a thick needle, he gently and slowly forces the anesthetic into the cancellous tissues. My first experience led me to confine both the preliminary and main injection to the tough portion of the gum, and I have had no reason to alter. My usual practice is to inject at a point just below the interdental papilla. For this I use the small size syringe I have already mentioned, with an ordinary solid hub needle ground down to about one-eighth of an inch in length, and having a rather short bevel of about forty degrees. Having injected into the gum, with a fine spear or chisel-shaped drill of the same or only slightly greater diameter than the needle, I pierce the cortex. Using the same needle and syringe and pressing it home till the hub rests against the bone, I gently inject from five to ten minims of two per cent. novocaine. This procedure I find to be thoroughly satisfactory. The open character of the cancellous tissue allows of the rapid diffusion of the drug, and, although injected about a quarter of an inch further from the apex than Parrott advocates, the anesthesia is almost invariably immediate. I consider this technique to have several advantages. It is somewhat simpler and simplicity tends towards success. There is only one small puncture, and, whie the bone is pierced at a point where the septum is somewhat thinner than it is nearer to the apex, that difference is small and is counter-balanced by the thinner drill used and the greater exactness with which one can judge the position of the septum. There is only one needle used, thus avoiding changing and handling, and with the small light syringe there is good control. Owing to the open and vascular nature of the interior of the bone, little pressure is usually required to inject. If the plunger does not move with ease, it means that the drill has not perforated the outer dense layer, or that the needle is blocked by a spicule of bone. The needle must be withdrawn and cleared, or the canal deepened, as the case may be. In young persons it is often unnecessary to use the drill to obtain access, as the needle can in a great many cases be forced through the cortex without difficulty. The lumen of the needle is liable to be blocked, but by having a fairly long bevel and slightly turning the point over, this to a great extent can be prevented.

To intra-alveolar injection there are two disadvantages. At the moment of injection there is sometimes some degree of discomfort felt by the patient—the heart's action becomes excited and the patient has a feeling of faintness or of oppression. This is accompanied by blanching of the face and lips. These symptoms, however, are transient, lasting only for a few seconds, as a rule, and are mainly referable to the action of the vasoconstrictor, some of the anesthetic being forced directly into the circulation owing to the unyielding nature of the bony walls, so preventing that elastic yielding of the tissues which obtains in more open localities. It is advisable, therefore, to reduce the proportion of suprarium.

By using the saccharine corporation tablets and combining one or two E and one F, these symptoms are lessened. In young persons these symptoms seldom appear. It is advisable, before beginning, to warn patients that they may have an unpleasant sensation, but that it is transient, as there can be no doubt that the mental attitude of the patient greatly influences the condition.

The second disadvantage is that there is sometimes slight after-uneasiness, seldom amounting to actual pain, and usually only admitted by the patient on inquiry. This is probably due to the reaction within the bone-enclosed cavity which on an external part would not be sensible.

Regional anesthesia, so far as it is usually applied in dental work, is not so formidable or difficult as might at first appear. It has several outstanding advantages over other methods. In conservative work, where one will usually be dealing with one area at a time, only one puncture as a rule is necessary. The tissues through which the needle has to pass are practically insensitive, and at the point of deposition of the anesthetic are of an open, unresisting nature, so that there is no sensation of pressure. From this open nature of the parts the drug is gradually absorbed and immediate reflex symptoms are therefore absent. The only objection to this method as compared with others is the length of time which elapses before anesthesia is complete. Where several teeth are to be prepared this is fully compensated by the length of time at one's disposal for doing so.

There are in practice four regions on either side by which to induce anesthesia: the post maxillary and infra-orbital regions in the upper jaw and the mandibular and mental in the lower. To make satisfactory and intelligent use of this method it is necessary to have a thorough knowledge of the disposition of the parts, particularly of the nerve supply. By means of a few slides I shall endeavor to indicate the main features rather than by oral description. For the technique, it has been so fully explained in the various treatises, that I can not do better than refer you to them. The only departure I would suggest is the elimination of bayonet attachments, as my experience is that they are unnecessary, and a straight instrument is under better command if it can be used.

With scrupulous antiseptic preparation, local anesthesia in conservative work is not only safe and simple, but by the steadiness of the patient conduces both to comfort and a higher standard of work. Unlike other methods of obtunding dentine, there need be no fear of causing inflammation and death of the pulp, as the action of the drug is entirely outside of the tooth itself.—*The Dental Record.*

